# Escaping the Laboratory: An Escape Room to Reinforce Biomedical Engineering Skills

**DOI:** 10.1007/s43683-022-00089-w

**Published:** 2022-11-03

**Authors:** Sunny Kwok, Rachel Childers

**Affiliations:** Department of Biomedical Engineering, The Ohio State University, 4100B Fontana Laboratories, 140 W 19th Ave, Columbus, OH 43210-1110 USA

**Keywords:** Escape room, Game-based learning, Active learning, Student engagement, Laboratory

## Abstract

Commercial escape rooms have grown in popularity as an enjoyable experience that also doubles as an exercise in communication and collaboration. Educators can take advantage of these natural qualities to engage and support students in a low-stress learning environment. The primary goal of this study is to share the development and application of an educational escape room as a tool to provide biomedical engineering (BME) students with an immersive and practical experience. A BME laboratory course-specific escape room was developed and beta-tested on an initial group of BME students. The first set of feedback enabled improvements to the design and difficulty of the escape room, which was followed by the final release of the activity for the intended undergraduate BME course. Across an academic year, 74 participants agreed to provide survey feedback for this study. Despite a moderate escape rate (29%), students reported high satisfaction and enthusiasm for the activity. Student survey responses indicated that participants were engaged and empowered to successfully escape even without external motivators. Responses supported the effectiveness of the escape room as a BME learning environment, allowing students to practice and retain course-related knowledge in a challenging but low-risk activity. The foundational structure of escape rooms offers a beneficial environment for experiential knowledge application. We conclude that educational escape rooms show promise as a pedagogical tool in promoting enhanced knowledge retention through immersive, game-based learning.

**Supplementary Information:**

The online version contains supplementary material available at 10.1007/s43683-022-00089-w.

## Introduction

Escape rooms have been growing in popularity since the early versions established in Japan in 2007; prior to the COVID-19 pandemic, their popularity had peaked to over 2,250 escape rooms in the United States.^42^ In the classroom, escape rooms have gained attention across all levels of education including primary, secondary, and especially in higher education^11,17,40,43^ as an immersive learning experience. Escape rooms are a narrative-based challenge that requires completing a series of puzzles or tasks with a small group of people in a limited amount of time. Successful completion of escape rooms results in either escaping from a physical room or completing a final objective such as breaking into a vault. Escape rooms have been touted as excellent team building exercises and reward creativity, leadership, communication, and critical thinking.^32,33^

With the opportunity to practice these skills, it is not surprising that some educators have adopted escape rooms themed to also teach domain-specific knowledge. Escape rooms for K-12 classrooms may cover a wide array of subjects. One such virtual escape room game was developed to allow the educator to select from subjects ranging from history and English to chemistry and mathematics.^11^ In higher education, most escape rooms tend to be in either healthcare professions or STEM related disciplines.^40^ Escape rooms have been implemented in a variety of fields including medicine,^1,21,24,44^ engineering,^12,13,34,35^ and others.^20,26,43^ Due to the versatility of its structure, different formats of escape room have been used to target specific learning outcomes. Table-top breakout boxes consisting of a series of puzzles in a box was designed to teach genetics concepts to undergraduates^8^ or digital electronics skills to second-year students.^35^ Aspects of gamification combined with the escape room format have been used to create a virtual escape room through MATLAB to prepare students to address bioimaging course outcomes in biomedical engineering (BME).^22^ Virtual and table-top escape rooms are valuable in that they may be easier to scale for large courses. However, physical escape rooms are more immersive, may be more motivating for students, and seem to better facilitate communication and collaboration.^2^ In this innovation article, we describe the first, to our knowledge, physical BME focused educational escape room.

An escape room can be a low-stress alternative for practical laboratory examinations where students are expected to successfully demonstrate technical skills. Gamification has been identified as a potential method to reduce testing anxiety.^38^ In BME, game-based learning has been used to create a low-stress environment in midterms and exam review sessions to decrease test anxiety in an undergraduate Biofluid Mechanics course.^6^ Incorporated into the design of an educational escape room, students are required to apply domain-specific knowledge in response to problems presented throughout the activity. Furthermore, a physical escape room lends itself well to testing students’ abilities to use equipment or perform technical tasks like those that might be learned in a laboratory course. Several educational escape rooms have seen successful implementation in clinical fields. For instance, an escape room was used to measure whether nursing students retained information from didactic lectures to complete different skill tasks, where progression was not allowed until a moderator observed the skills completed successfully.^1^ Another escape room, designed for medical school students, demonstrated that the experience helped motivate students to prepare in advance and retain knowledge necessary to complete tasks related to vascular surgery.^24^ In each case, the escape room activity required real-time application of background knowledge and execution of physical skills.

A variety of techniques have been explored to grade escape room activities including through attendance and participation,^27^ point systems related to the fastest clear times,^21^ or grading schemes related to specific learning objectives.^43^ However, only about one third of escape rooms report assessing students or teams during the escape room.^43^ Whether or not students are graded in the escape room, several other study authors have pointed out the necessity to provide feedback to students about the experience through a debrief.^4,18,19^ That is, feedback to the students about their learning does not necessarily need to happen in the form of a grade.

A grade may not be necessary to motivate student participation in escape room activities. Gamification, using aspects of a game to increase engagement, has been a method to increase student engagement and motivation in various aspects of education.^5,14^ Educational escape rooms take advantage of this concept to gamify and strengthen student learning outcomes.^7^ In one study, chemical and industrial engineering students were more motivated to study heat transfer course concepts in preparation for an escape room experience.^12^ Similarly, in another study, computer science students also reported increased motivation to study with accompanying increase in learning gains measured by pre-tests and post-tests given outside of the escape room.^28^ That study also linked increased learning gains with an increase in the number of puzzles solved by the student during the escape room.^28^ One key aspect of gamified learning is promoting education through intrinsic motivators (e.g., curiosity, enjoyment, satisfaction, and interest) as opposed to reliance on extrinsic motivators, such as a grade.^9^ Self-determination theory (SDT) identifies areas of intrinsic motivation such as the needs for competency and autonomy that are essential to human social and personal well-being.^36^ SDT provides compelling evidence linking intrinsic motivators, such as autonomy and competence, to both increased academic achievement^41^ and enjoyment of gaming.^37^

One challenge for both gaming and education lies in finding the right balance between an activity’s difficulty and the skill of the participants. An easy task may result in lowered participant satisfaction and boredom; in contrast, an overly difficult task may lead to frustration and anxiety. Proper challenge-skill balance has been shown to enable a motivating state of “flow” when there is an appropriate level of challenge to match a person’s skill.^16^ In the field of positive psychology, this optimal state of focused concentration resulting from proper matching of challenge and skill is referred to as flow theory.^31^ Flow theory has been studied under the scope of education, revealing strong correlations between the state of flow and heightened motivation, self-efficacy, and satisfaction.^23^ For an escape room, one way to modulate the difficulty of an experience in real-time would be through intervention in the form of hints or feedback. Commercial escape rooms utilize a “Game Master” (GM), who initially introduces the narrative and then serves as a resource for hints if necessary.^32^ In educational escape rooms, the GM may be an instructor or teaching assistant who facilitates learning through game design or through clues and hints to guide the learner. The GM may intervene when appropriate to help students navigate a particularly difficult obstacle but must also keep in mind the potential of providing excessive assistance resulting in reduced student ownership of success. As with any other learning experience, optimization of the challenge level of an escape room is crucial to enable the potential benefits it may provide as an educational tool.

Here we describe the design, implementation, difficulty adjustment, and results from an in-person, physical biomedical engineering laboratory escape room. The learning objectives of this escape room were: (1) to evaluate the retention of course material, (2) provide a practical setting to apply and reinforce newly acquired laboratory techniques, and (3) to encourage teamwork and communication skills in advance of a group project.

## Methods

### Course Structure

The escape room was designed for a semester-long upper-level BME laboratory course (typically third-year students). The laboratory course teaches a variety of BME lab skills across broad topics. In the first 6 weeks of the lab, students build technical and laboratory skills such as micropipetting, aseptic technique, and cell culture skills. They learn to use and analyze data from equipment such as microscopes, spectrophotometer plate readers, uniaxial mechanical test frames, and ultrasounds. The escape room is situated in the 7th week of the semester, preceding an open-ended final project where students design experiments to address a hypothesis of their own. The escape room provides an opportunity for the students to review laboratory skills and practice with equipment before they work on final projects in small groups of 2–4 students. In the beginning of the semester, students were made aware that the escape room would occur in the 7th week and that skills from the entire semester would be needed to complete it successfully.

### Escape Room Development

The room was designed to be completed in groups of 8 or fewer participants and within 45 min. A time limit of 45 min was originally selected to facilitate scalability and to allow multiple groups to move through the escape room within a 130-min course timeframe scheduled for each laboratory section. This provides enough time to allow two groups to move through the escape room, including a short reminder about rules and presentation of the objective/narrative before the experience, and a short debrief for each group after the experience. It also allows for the instructors to turn over the room (reset puzzles, locks, etc.) in between the two groups. This setup permitted up to 64 students to participate in a single escape room across 4 different lab sections all within one week of the semester. Group sizes were targeted to be between 6 and 8 people because this size is small enough that all students will be able to actively participate on at least one puzzle or task at almost all times during the 45-min window. In addition, it is still within range of what has been described as acceptable by other educational escape room designers.^43^

**Figure 1:**
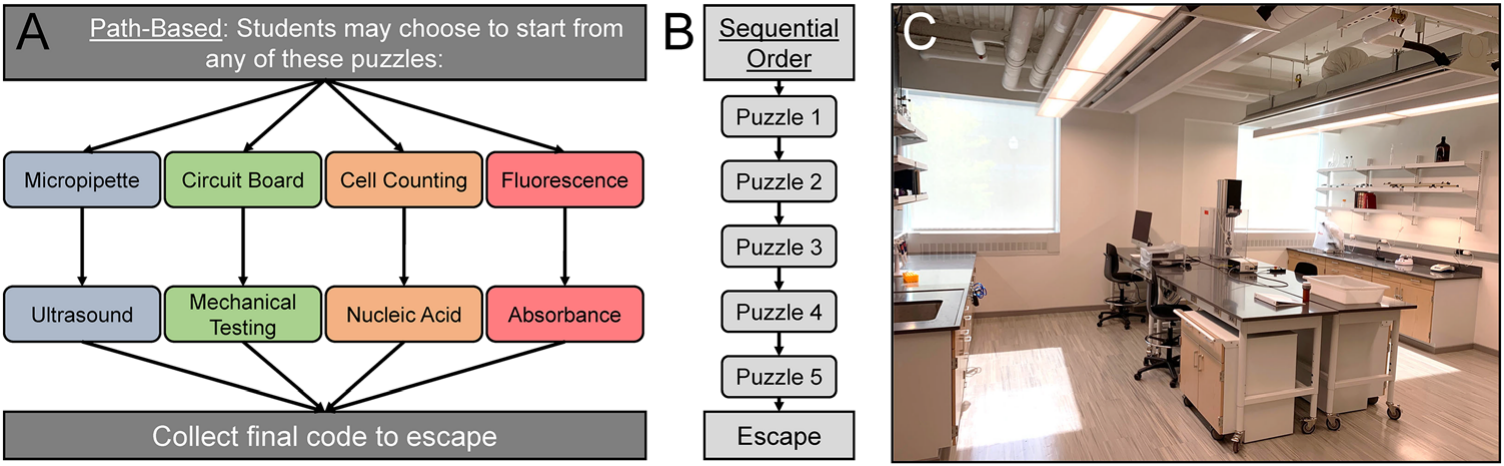
(a) The BME lab course escape room was designed to provide students with a non-sequential, path-based structure of puzzles to encourage teamwork and collaboration. Students start with all the necessary materials to engage with four different puzzles in parallel. (b) An example of a sequential design of escape rooms (c) Immersive room design encourages student learning within a new but familiar environment.

During the planning of the escape room, a primary goal was to incorporate the major laboratory techniques explored throughout the course, which included (1) using a spectrophotometer and analyzing standard curves, (2) micropipetting, (3) cell counting and cell density calculations, (4) dynamic mechanical testing, and (5) ultrasound imaging. Additionally, other biomedical engineering-related background was included as appropriate to the plotline. Each of these techniques were integrated into a puzzle and organized into four pathways of the escape room (Fig. 1a) in which completion of each pathway leads to one digit of the final escape code (a vial containing the sought-after cure). This organization of puzzles into multiple paths is called “path-based”.^32^ Often a sequential path for an escape room is selected because it is simpler to monitor, and easier for students to identify how to progress.^43^ However, a sequential path escape room (Fig. 1b) creates barriers to entry, requiring students to figure out clues in series before they can progress. Even though a sequential path is the most popular design among educational escape rooms,^43^ we wanted to create options for exploration as well as prevent bottlenecks at puzzles. Therefore, we selected the path-based design to allow more students to be simultaneously engaged in puzzle-solving and increase the need for social interdependence. The path-based design allows students to explore the environment and rotate to other puzzles if they are unsuccessful on a specific puzzle, reducing the potential for frustration and/or reduced motivation. Not being stopped at one puzzle allows participants to step away from the puzzle that may be an obstacle to them and work on other aspects of the room that can be completed successfully. In addition, the path-based design provides several routes for a successful escape. For example, if the participants are unable to complete one puzzle sequence, they may still be able to successfully escape by attempting to guess the corresponding digit on the final lock code.

The escape room was introduced with a narrative to create an immersive experience. The short narrative was delivered before the students entered the space: “Eight of our top scientists have been working on therapies and technology for a new [fictitious] strain, COVID-∞. We recently received information that an effective new cure has been under development! However, a recent breach of security by an unknown infiltrator in the laboratory resulted in our scientists falling ill as well as major disruption to the laboratory. The scientists have since been forced to quarantine. Although the agent was captured by our operatives, they still managed to sabotage the research done by our scientists. We’ve asked for several BME experts to rediscover the cure by undoing the sabotage.”

**Figure 2:**
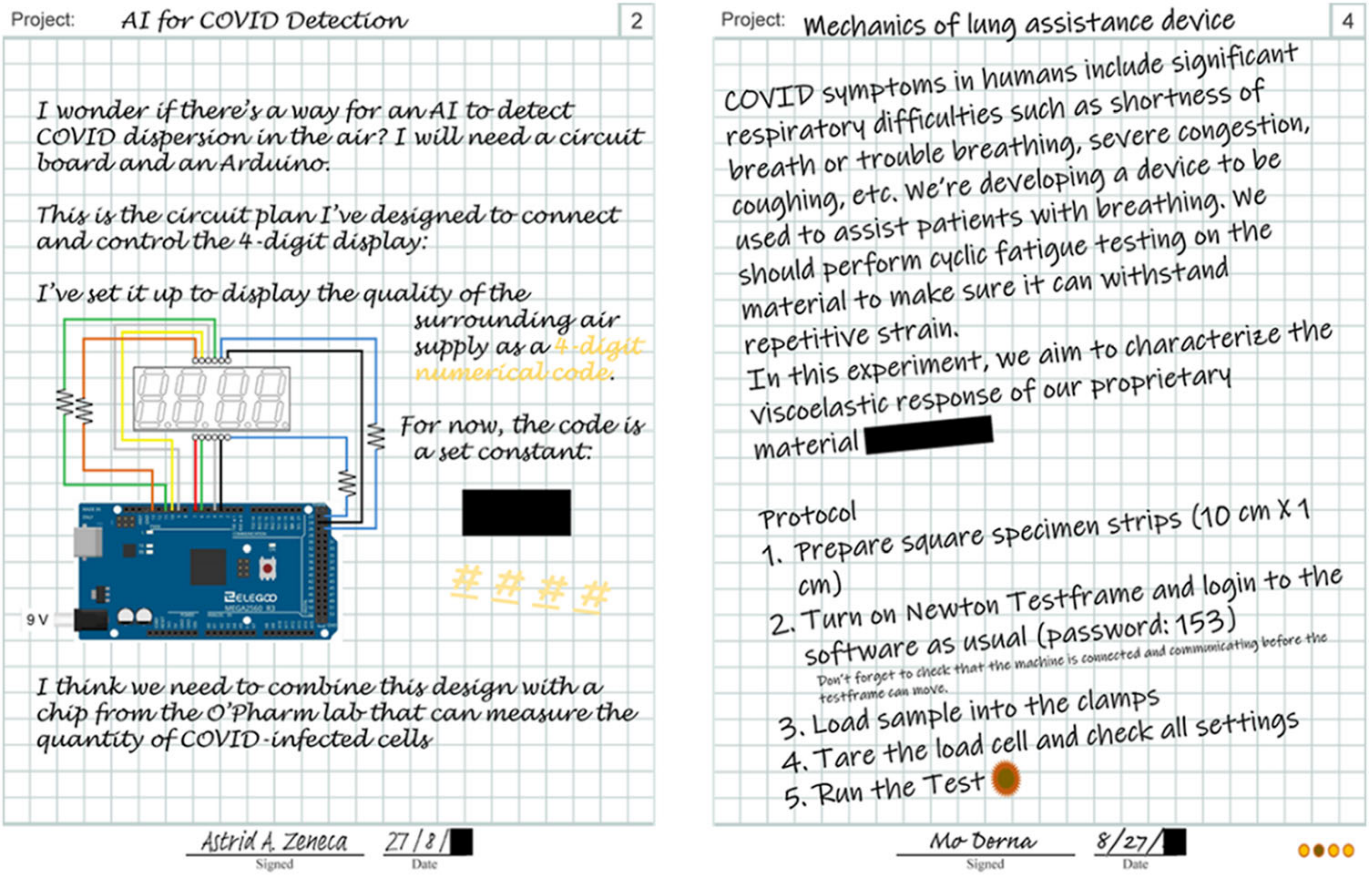
Sample journal pages including necessary hints to solve puzzles complemented by nonessential narrative elements to establish an immersive environment.

Eight lab notebook pages were created that corresponded to each of the quarantined scientists. These provided students with both the objective and context of the puzzles. A sample of notebook pages can be seen in Fig. 2. Students were provided with a binder containing a few of the notebook pages at the start, while the rest were separated and hidden throughout the room. Essential tools and equipment were placed around the room, which was decorated to resemble a sabotaged lab space (Fig. 1b).

In one pathway, students must apply their micropipetting skills to accurately estimate unknown volumes of solution. This value from the micropipetting challenge is then used to unlock another piece of essential equipment. Other parts of equipment need to be unlocked by correctly performing a calculation they are familiar with from previous labs. With the path-based organization of puzzles, students may need to complete a first puzzle as a gateway to the second. For instance, in order to use an ultrasound, the key to unlock a cabinet containing the transducer needs to be recovered. The ultrasound can then be used to image a phantom which provides the result as a digit of the final escape code. A careful plan of placement of each item is necessary to ensure puzzles can be completed in a specific order. In addition, due to the complexity of the path-based organization, coding is used to indicate puzzles and necessary clues. For example, color-coded stickers are placed on notebook pages and their corresponding lock.

**Figure 3:**
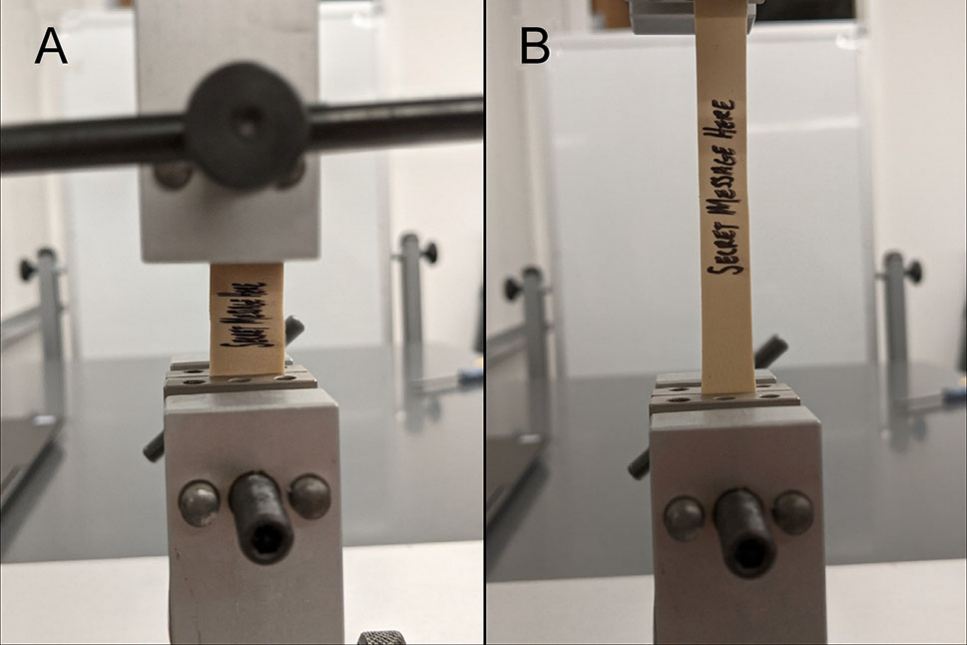
Example mechanical testing puzzle (a) before and (b) after solving to uncover a secret message important for successful escape.

In another pathway, students must follow the circuitry design in a lab notebook page to complete a disassembled circuit board. The repaired circuit board will display a four-number code that unlocks a cabinet containing the mouse and keyboard for the computer system in the room. The computer is essential for the operation of both the Newton mechanical testing device and the Biotek plate reader. The students must successfully operate the machine to stretch a rubber band already loaded into the machine but not under tension. The stretched rubber band will reveal a message for the final digit (Fig. 3). This puzzle also requires collaboration as the table the mechanical test frame is on is “contaminated” and situated far away from the computer where the operator would not be able to read the message by themselves. In addition, any student who touches the contaminated table is penalized with an oversized glove (to “decontaminate” them). This requires that more than one student work on the puzzle to solve it due to the physical separation of the mechanical test frame and the computer in the lab space.

In other puzzle pathways, students perform various tasks to complete puzzles such as calculate cell density of a simulated hemocytometer loaded with cell solution, which was created from a custom printed film (Gamma Tech, Albuquerque, NM) and mounted on a microscope slide. Another puzzle is to match solution density based on absorbance readings using a spectrophotometer after pipetting standards and unknowns into a 96-well plate. Entering all four numbers in the right sequence into a bottle labeled as “The Cure” before the 45-min timer runs out dictates a successful escape. Importantly, the four paths enable students to miss the mark on one set of puzzles and still successfully guess the last digit of the escape code.

We employed an iterative process during the escape room prototyping phase to continually assess the robustness and difficulty of the puzzles. The goal was to create a set of challenging puzzles that were appropriate in difficulty to match student skill level, targeting the optimal state of flow to maximize engagement.^25^ Each puzzle was created and tested independent of one another, and the connection between puzzles can be established with the resulting codes or keys at the end. This provided the freedom for the puzzles to be shifted in sequence if needed. During this process, a few puzzles were simplified or altered for clarity. For example, one puzzle required participants to calculate the concentration of an unknown sample using absorbance. The concentration result was a code entered into a lock, but this required participants to find the exact concentration. For the simplified version, we printed a table with sample IDs and concentrations then attached it to the wall. Instead of entering the concentration, students would enter the sample ID as the code. This allowed some room for variability or error in measuring concentration, so if they were in range of the true value, they could identify the correct sample ID as the code for the lock. In another case, we simplified puzzle elements such as the design of the circuit-board to reduce the complexity and speed up the solve-time.

### Escape Room Prototype Testing

The primary goals of the prototype (or beta-test) were to assess the difficulty of the tasks and effectiveness of the escape room for collaboration. Testers consisting of both graduate and upper-level undergraduate students were recruited to participate in the prototype escape room in groups of 4-6 participants. Before attempting the activity, participants were instructed to view an informational video about tips for escape rooms.^3^ Upon arrival, testers were given the expositional narrative as described above and provided with the general rules prior to entering the escape room. An instructor was also present in the area to moderate the activity. The instructor kept the time, documented observations, and would provide free clues when requested by the entire group. For the beta tests, participants were allowed to continue past the allotted 45 min if necessary to experience and provide feedback for all components of the escape room. After completion of the activity, an anonymous survey was sent electronically to the beta testers.

### Escape Room Implementation

After making several adjustments to the escape room in response to the results from the prototype escape room tests, the improved activity was incorporated into the BME laboratory course curriculum. This activity was scheduled after six weeks of laboratory course instruction but before the final group research projects. Students were assigned to groups of 2 to 4 for their final group projects, which were combined into teams of 6 to 8 participants for the escape room activity. This provided students with the opportunity to work with potentially unfamiliar classmates and/or further develop their teamwork and communication skills with their project groups. Keeping the format of the beta test, students were given the expositional narrative and provided the general rules prior to entering the escape room. Students were also informed that their efforts will be quantified into a group score at the end of the activity, and this will be displayed on a class leaderboard (Eq. 1). The group score on the leaderboard was added as a feedback-giving mechanism. Leaderboards are a gamification technique that can provide an aspect of enjoyment and potential motivation.^39^ The leaderboard did not affect their grade but served primarily as social feedback with group names and corresponding scores displayed during class. An instructor was present to keep the time and document observations. From the prototype feedback, it was decided that students would be allowed to explore the space for 15 min first, at which point the instructor would provide a free clue every 5 min until the timer ends (a total of 5 free clues). The students were encouraged to ask for more clues as a group, however any additional clues will factor into the group score.

1 $${\text{Score }} = \, \left[ {{\text{Time }}\left( {{\text{min}}} \right)} \right] \, + \, \left[ {\# {\text{ of Extra Clues}}} \right] \, - \, \left[ {{3 }* \, \# {\text{ of Puzzles Solved}}} \right] \, - \, \left[ {{4 }*{\text{ Cure Obtained}}:{\text{ Yes}}\left( {1} \right)/{\text{No}}\left( 0 \right)} \right]$$

### Escape Room Assessment and Participation

Instructors observed students during the completion of the activity and recorded observations about number of clues needed, which puzzles were solved, and timing of completing certain achievements. After completion of the activity, an anonymous survey was sent electronically to participants (Supplementary Information 1). The survey questions were adapted from four other studies on educational escape rooms.^1,12,24,34^ Student rating of the difficulty of various escape room puzzles were converted to a 3-point Likert scale (Easy—1, Moderate—2, Difficult—3) for relative quantitative comparison of difficulty between beta testers and students in the course. Student responses to other survey questions were tabulated to describe the level of agreement expressed for each statement. Students’ responses to open-ended questions were reviewed for relevant themes including satisfaction, motivation, teamwork, and learning. The survey questionnaire and methodology for this study was approved by the Institutional Review Board of the The Ohio State University and determined as exempt (study ID 2022E0178). Informed consent was obtained from all individual participants included in the study. A total of n=64 undergraduate students (*n* = 30 in Fall ’21 and *n* = 34 in Spring ‘22) who were enrolled in the upper-level course (mostly juniors) agreed to participate in the survey. An additional *n* = 10 responses were collected from beta testers who were a mix of graduate and undergraduate students who had previously taken the course, but not participated in the escape room before or graduate students in the department. All participants were confirmed to be at least 18 years old. While demographic data was not collected as part of the study, the students are expected to be representative of the Biomedical Engineering department as a whole since this the escape room was placed in a required course and all undergraduate and graduate students were from the BME department. Between 2019 and 2022, 46% of BME students identify as female, and 8% of students identify as Black, African American, Hispanic, American Indian, Native Hawaiian, or Pacific Islander.

## Results/Discussion

### Prototype Test Results

Two groups (*n* = 12 total) participated in the prototype (“beta-test”) escape room and 10 participants agreed to provide survey feedback. All prototype testing participants were teaching assistants at the graduate or undergraduate level. Only 40% of the beta-test participants had participated in an escape room experience prior to this one and 50% of the participants viewed the instructional video. Between the two groups, it took an average of 54 min to successfully escape, and neither beta-test group successfully completed the activity within the allotted time of 45 min. Between the two groups, participants asked for an average of 5 clues to complete the activity.

Participants were asked to provide feedback for the prototype escape room after completion of the activity. 100% of participants agreed/strongly agreed that this activity encouraged collaboration between team members. Furthermore, 90% of participants agreed/strongly agreed that this activity encouraged communication between team members and encouraged the use of leadership skills. These responses support the design choice of a multi-pathway escape room layout to promote student teamwork and collaboration.

**Figure 4:**
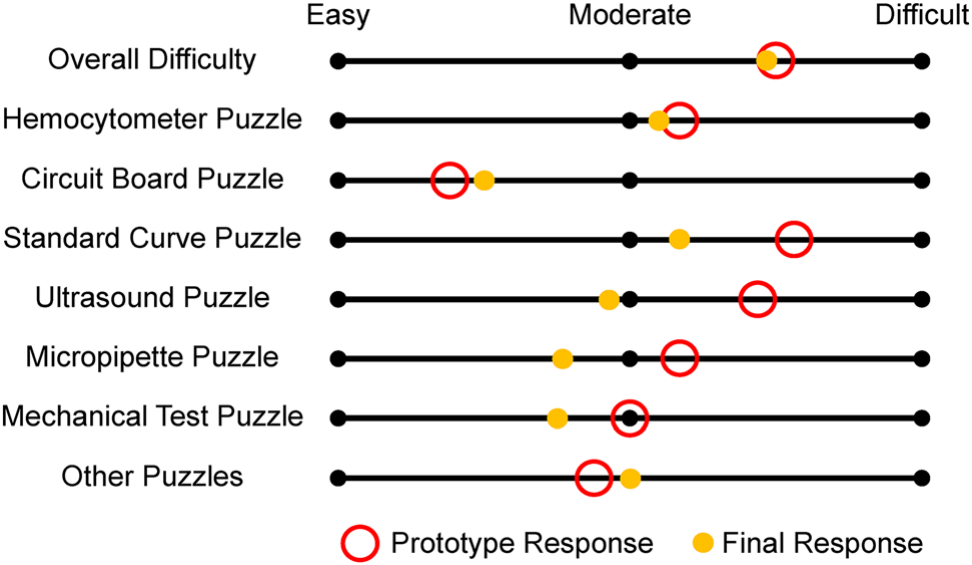
Average student rating of the difficulty level for each major component of the escape room during the prototyping stage (red open circle, *n* = 10) and the finalized escape room implemented in the course (yellow filled circle, *n* = 64). For most of the puzzles, the difficulty of the activity was made easier for the full release. “Other Puzzles” refers to all other minor activities such as reading notebook pages, minor puzzles, and finding keys.

Figure 4 shows the feedback from participants in the prototype version vs. participants in the final version implemented in the course, regarding the difficulty of the escape room puzzles. Beta-test participants also offered feedback on methods of improvement. Almost all student responses included recommendations for either increasing the allotted time for the event or reducing the difficulty for certain tasks. These suggestions influenced the restructuring and/or redesigning of some of the tasks that were rated the hardest difficulty by the participants. The instructors also observed that the participants often appeared hesitant to ask for clues, with the highest volume of requests being condensed into the last 5 min. Therefore, the adoption of a time-controlled and free clue system was expected to decrease the difficulty of the activity and encourage timely progress. Lastly, a few beta-testers provided feedback suggesting a reworking of the instructional notebook pages. The content of these pages was streamlined to reduce the necessary reading time and increase visibility of the objective.

### Final Escape Room Results

**Figure 5:**
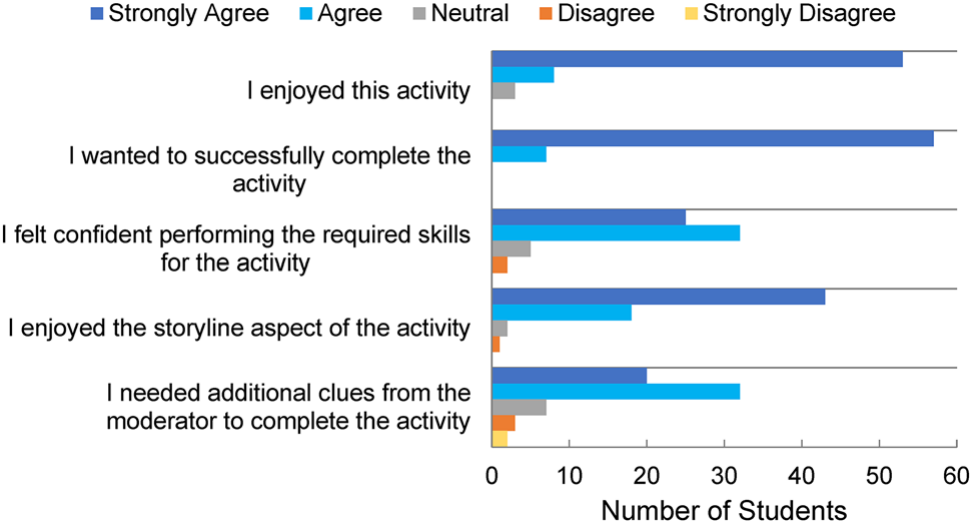
Survey questions pertaining to student satisfaction after completing the final escape room. Almost all students reported a positive experience and a desire to successfully escape.

Across two semesters of this course, a total of fourteen groups of students (n = 92) experienced the escape room, of which 64 students agreed to participate in the survey. Four groups were able to successfully escape within the allotted time, yielding a success rate of 29% (compared to roughly 41% for commercial escape rooms worldwide or a 26% success rate in North/South America^32^). The fastest time was one group that completed the room in less than 36 min. When asked to rate their enjoyment of this activity, 95% of students answered agree/strongly agree (Fig. 5). Interestingly, although this activity was not graded for successful escape, 100% of students agreed/strongly agreed toward wanting to successfully complete the activity. This suggests that students were engaged in the activity, which may lead to greater personal investment in their education. Furthermore, 89% of students agreed/strongly agreed that they felt confident performing the required skills for the escape room puzzles. When asked if they would prefer a traditional lab practical to the escape room, 95% of students would prefer the escape room format. These responses suggest that testing student knowledge with the escape room format may empower students and reinforce student retention of the course material.

**Figure 6:**
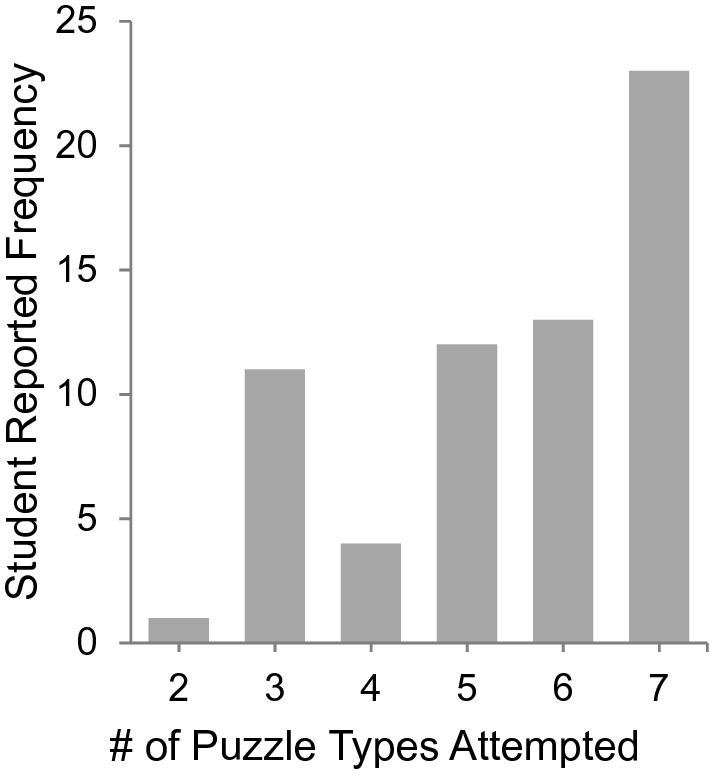
The path-based escape room design enabled students to work on many puzzles in parallel. A majority of students reported attempting most, if not all of the puzzles during the activity. Puzzle types included six course-specific tasks and “Other Puzzles” which consisted of BME domain-specific knowledge.

The multiple path-based design provided students more freedom to explore and more possible ways toward collaboration and successful escape. Since the escape room has several puzzles that address a variety of laboratory techniques and BME domain knowledge, we looked at survey responses to evaluate how many puzzles each student reported attempting. Puzzle types include the six course specific tasks and one “Other Puzzles” category which consisted of all other puzzles requiring BME domain-specific knowledge. All students attempted at least 2 different puzzle types, 70% of participants reported attempting all 7 puzzle types. On average, students reported engaging with 5.5 puzzle types out of 7 categories (Fig. 6). This is consistent with the instructor’s observations that students engaged with many different puzzles, and collaborated with other individuals to complete tasks.

**Figure 7:**
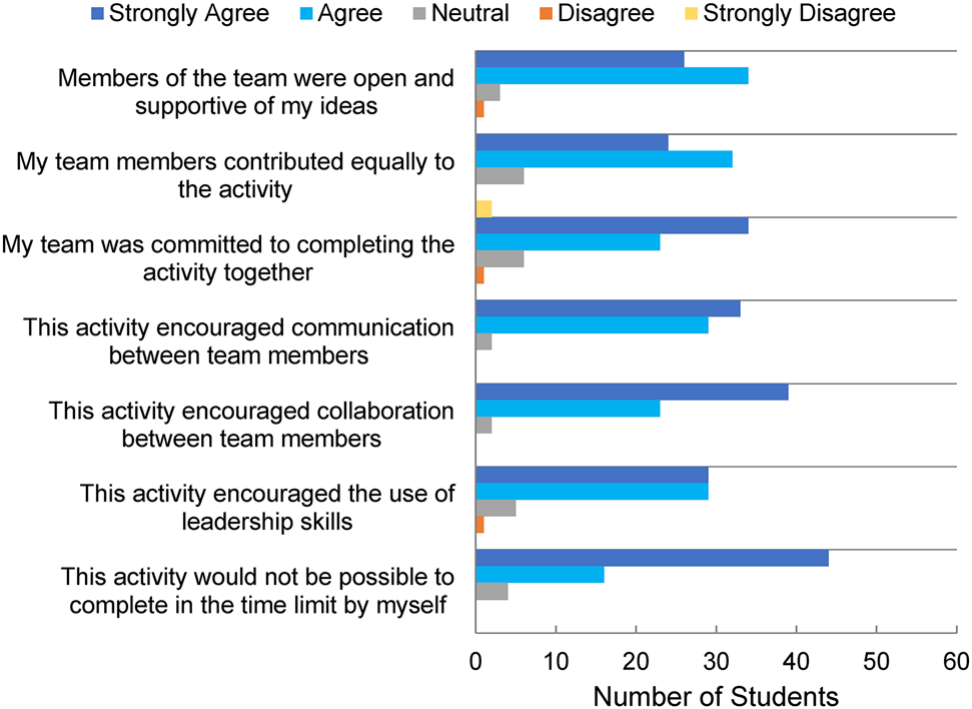
Survey questions pertaining to effective teamwork and group dynamics during the escape room activity. Results support the effectiveness of escape rooms in promoting communication, leadership, and collaboration.

Escape rooms, like many engineering occupations, require collaborative effort and as such, student teamwork and communication within the escape room activity were also evaluated. Similar to the responses from the prototyping stage, an overwhelming majority of the students agreed/strongly agreed that the activity encouraged communication and collaboration between team members (97% for both, see Fig. 7). 94% of students agreed/strongly agreed that they felt supported by team members, and 88% students agreed/strongly agreed that team members contributed equally to the activity. For 91% of students, they agreed/strongly agreed that the escape room encouraged the use of leadership skills. One student commented that their favorite part of the escape room was “working with my lab mates, getting to stretch my leadership skills for the first time in a few years, and getting out with time to spare.” Overall, 94% of students agreed/strongly agreed that this activity would not be possible to complete within the time limit by an individual. It is important to acknowledge that the choice to design a multiple pathway-based escape room necessitates effective student communication and collaboration for a successful escape. When asked to describe the best part of the escape room experience, 20 respondents specifically identified “collaboration” or “teamwork” within their free-response answer. One student stated that the best part of their escape room experience was “working with others while having a goal everyone actually cared about [because] in most group [projects], people don't actually care.” Another student described “the best parts of this experience was meeting new people and working with classmates towards a common, tangible goal.” Combined with the student responses indicating a high level of satisfaction toward the activity, this further supports the value of the escape room format as an opportunity for students to collaborate and practice communication and teamwork skills.

**Figure 8:**
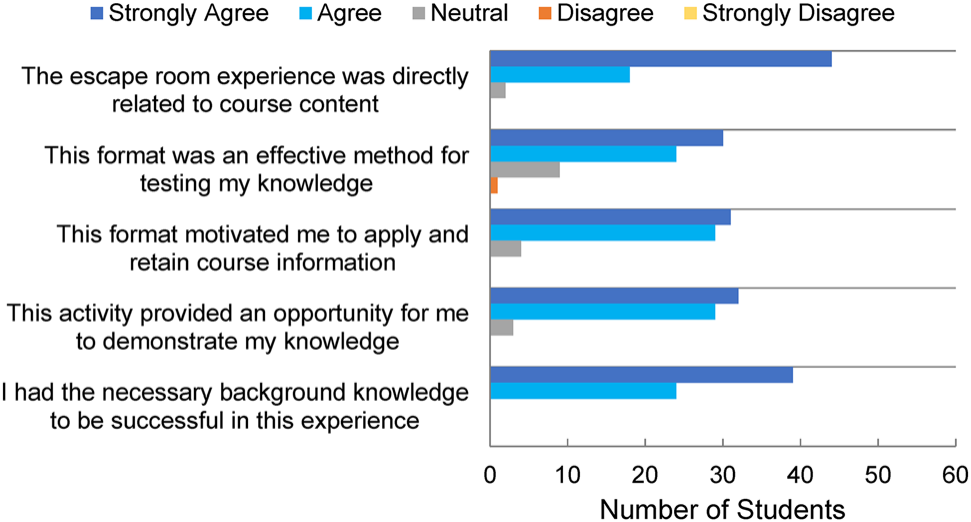
Survey questions pertaining to the effectiveness of the escape room in assessing student learning objectives. Students’ responses suggest a high level of knowledge retention and an effective environment to apply course material.

Students were also asked to rate their level of agreement on several survey questions assessing the efficacy of the escape room activity in addressing and assessing course learning objectives (see Fig. 8). 97% of students agreed/strongly agreed that the escape room activity was directly related to the course content and 100% of students agreed/strongly agreed that they had the necessary background knowledge to be successful in this experience. These responses indicate that the activity successfully complemented the laboratory course material. The survey also asked the students to rate if this escape room format was an effective method for testing their knowledge, of which 54 out of 64 students agreed/strongly agreed with the statement. 94% of students agreed/strongly agreed that the escape room format motivated them to apply and retain course information, as well as presented an opportunity for them to demonstrate their knowledge of course material. Applying course skills was also mentioned by 17 respondents in a free response to what were the best parts of the escape room experience. Overall, student responses demonstrate the effectiveness of the escape room format as a practical setting to apply laboratory techniques previously learned through the course.

As seen in Fig. 4, the full escape room experience was rated to have similar overall difficulty as the prototype, that being in between moderate and difficult. In spite of this, many of the updated puzzles were rated to have reduced difficulty on average compared to the prototype responses. On top of the five free clues provided by the instructors, the students asked for an average of 1.9 additional clues across all groups with some groups needing as many as 4 and two groups not asking for any. From the survey, 81% of students agreed that additional clues were necessary to complete the activity (Fig. 5). Within the post-activity survey, students were also given the opportunity to provide feedback on methods of improvement. Out of the 51 responses, 21 comments suggested methods for simplifications/clarifications and 10 comments mentioned extending the time limit.

One challenge of the design of the escape room was in creating the appropriate difficulty level while still making a successful escape possible in 45 min. After the prototyping stage, it was decided that a clue system could help make sure participants were progressing. In line with the backstory, the clues were disguised as information reported by an intelligence agency from their interview with the captured saboteur. After an initial 15 min, clues were announced to the group every 5 min even if they were not requested. However, several comments from student participants in the post-survey suggested that the overall difficulty was not the issue, but they would rather have more time and less clues. One student said they wished they had “…60 min, instead of 45 would have allowed us to escape with less hints, and it’s less fun to need hints.” A couple of other students remarked that they appreciated that the escape room was challenging. Another student said, “I really enjoyed the difficulty level and the fact that we did not finish.” Taken together, this stresses the importance of empowering students with a challenging game design without providing too many clues, even if they do not successfully escape on time. This is consistent with SDT, which links the feeling of autonomy to enjoyment and motivation.^36^ In future implementations, less automatic clues should be provided, in order to not infringe on the sense of autonomy. Others have made similar observations in education escape rooms where students expressed their desire for more autonomy and less help from the instructor.^20^ An effective escape room should promote participant motivation and satisfaction, which may be achieved even without a successful escape.

## Conclusion

In this paper, we described the design process of a biomedical engineering-themed escape room that was incorporated into the curriculum for an upper-level undergraduate laboratory course. Using a path-based approach, the escape room featured four independent pathways consisting of a total of eight major puzzles. As a group, participants were tasked with finding and completing each puzzle to acquire an escape code within a 45-min time limit. Within this study, participants had an average successful escape rate of 29%. In spite of the modest success rate, survey responses from participants still maintained high levels of satisfaction, teamwork, and engagement throughout the activity.

Feedback from the survey suggests that students felt empowered to successfully escape even without external motivators such as grades, supporting the concepts put forth by SDT. Some students listed it as a relief that a successful escape was not necessary for their grade and appreciated the low-stakes aspect of the escape room compared to a traditional laboratory practical. Importantly, students indicated that they appreciated the opportunity to demonstrate their comprehension of course material. Others commented on the effectiveness of the activity as a way for them to build confidence. One student said, “It was a fun way to apply the skills we've learned in lab and showed me that I am more comfortable with these skills than I initially thought.” Others using educational escape rooms have also found that it is unnecessary to grade students in order to motivate and engage them.^43^

Through trial-and-error, the escape room structure and design were developed with a focus on an immersive experience that can also be an effective real-time application of course knowledge. During the prototyping stage, a significant hurdle was in establishing the difficulty level of the puzzles. As with any curriculum design, the scaling of the difficulty must be considered to optimize flow. Although the narrative simulated a high-level infectious disease research laboratory, the assessed laboratory techniques were much simpler by comparison. In an effort to increase immersion, the storyline and clue designs became hard to decipher and confused students with unnecessary details. As a result, the beta-version of the escape room was deemed too difficult by the initial group of participants. After streamlining various design elements and clarifying puzzle instructions, survey results indicated a more positive reception for the final-release escape room difficulty. Conversely, a new free-clue system was implemented with the final escape room as a method of scaling the difficulty in real-time. A few students suggested removing this system, one stated, “If possible, [maybe] extend the time to complete [in] 1 h so we don't have to get as many clues to finish within the time frame.” In consideration, providing free clues on a timer is akin to giving answers to students unprovoked. Many groups as a team agreed to ask the instructor for clues toward the end of the timer, but this is different from accepting free clues. We believe this system for scaling difficulty reduces student ownership and will require revising for future iterations of the escape room.

Significant future work is in development considering participant feedback and observed trends. First, further refinements of the puzzle difficulty and narrative elements are necessary. It was clear from the survey responses that many participants enjoyed the challenge of the escape room but wanted more time to successfully escape. According to one student, “I really enjoyed the difficulty level and the fact that we did not finish.” To address the feedback from participants, future iterations of the escape room will be expanded to 55 min instead of 45 min. Based on the feedbacks of our first beta-test, we successfully increased the escape rate from 0% up to the final 29%. We expect future iterations of a modified BME escape room to maintain this escape rate or provide students with even better odds of escape. Second, an important parameter that epitomizes the essence of an escape room is the value of teamwork. Effective communication skills and the capacity for collaboration are essential workforce soft skills that are built into the basic structure of an escape room. In the current study, the survey questions explored general aspects of group dynamics including communication, collaboration, and leadership that may be promoted by the escape room environment. It would be interesting to assess in greater detail the extent to which the escape room as an educational tool can promote and strengthen students’ communication skills and effectiveness within different group roles.

Escape rooms as a pedagogical tool show great promise for promoting enhanced knowledge retention through game-based learning. Importantly, although this specific escape room was tailor made for a biomedical engineering laboratory course, the versatility of an escape room-style activity lends itself to being widely applicable across a variety of subject domains.

### Citation Diversity Statement

Recent work in several fields of science has identified a bias in citation practices such that papers from women and other minority scholars are undercited relative to the number of papers in the field.^10,15,29,30^ We recognize this bias and have worked diligently to ensure that we are referencing appropriate papers with fair gender and racial author inclusion.

## Supplementary Information

Below is the link to the electronic supplementary material.

Supplementary file1 (DOCX 33 kb)
