# Supplementary material for: Escaping the Laboratory: An Escape Room to Reinforce Biomedical Engineering Skills
Source: Biomed Eng Educ. 2022 Nov 3;3(1):75–86. doi: 10.1007/s43683-022-00089-w (PMC9632588; doi:10.1007/s43683-022-00089-w)
Supplement: Supplementary file 1 — Supplementary file1 (DOCX 33 kb) [file 43683_2022_89_MOESM1_ESM.docx]

Supplemental Material 1: Survey: BME Lab Escape Room

1. Have you ever participated in an escape room before?
   1. No, never.
   2. Yes, but in a different format (e.g., board game or virtual)
   3. Yes, in a similar in-person format.
2. Did you watch the assigned YouTube video before participating in the escape room?
   1. Yes
   2. No
3. Did your group successfully complete the escape room in the time limit?
   1. Yes
   2. No
4. The time needed to complete the escape room (minutes)

|  | 0 | 5 | 10 | 15 | 20 | 25 | 30 | 35 | 40 | 45 | 50 | 55 | 60+ |
| --- | --- | --- | --- | --- | --- | --- | --- | --- | --- | --- | --- | --- | --- |

| Approximate time (minutes) () | 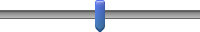 |
| --- | --- |

1. Please rate the level of your agreement with each statement below:

Strongly Disagree (1) Disagree (2) Neutral (3) Agree (4) Strongly Agree (5)

- I wanted to successfully complete the activity
- I enjoyed this activity
- I enjoyed the storyline aspect of the activity
- I needed additional clues from the moderator to complete the activity
- This format was an effective method for testing my knowledge
- I felt confident performing the required skills for the activity

1. Rank the difficulty level of the following parts of the escape room:

Easy (1) Moderate (2) Difficult (3) NA or Not Sure (0)

- The overall difficulty of the escape room.
- Hemocytometer/Microscope puzzle
- Circuit board puzzle
- Standard Curve/Absorbance measurement puzzle
- Ultrasound puzzle
- Micropipetting/liquid measurement puzzle
- Mechanical testing puzzle
- Other puzzles (lab notebook, nucleic acids)

1. Please rate the level of your agreement with each statement below:

Strongly Disagree (1) Disagree (2) Neutral (3) Agree (4) Strongly Agree (5)

- Members of the team were open and supportive of my ideas
- My team members contributed equally to the activity
- My team was committed to completing the activity together
- This activity encouraged communication between team members
- This activity encouraged collaboration between team members
- This activity encouraged the use of leadership skills
- This activity would not be possible to complete in the time limit by myself

1. Please rate the level of your agreement with each statement below:

Strongly Disagree (1) Disagree (2) Neutral (3) Agree (4) Strongly Agree (5)

- This format motivated me to apply and retain course information
- This activity provided an opportunity for me to demonstrate my knowledge
- The escape room experience was directly related to course content
- I had the necessary background knowledge to be successful in this experience.

1. Which answer describes your opinion best?
   1. I would have preferred a traditional individual lab practical exam to test my lab skills.
   2. Neutral. I don't have a preference between the escape room or lab practical exam.
   3. I prefer the escape room format

10) What recommendations do you have to improve the escape room experience?

11) What were the best parts of the escape room experience?
